# Collagen Matrix Implantation Following Pterygium Excision: Outcomes of a Preliminary Tested Hypothesis

**Published:** 2013

**Authors:** Muhammad Arish, Abdul-Nasser Nadjafi, Mojdeh Jahangard

**Affiliations:** Al-Zahra Eye Hospital, Zahedan University of Medical Sciences, Zahedan, Iran

**Keywords:** Collagen Matrix Implantation, Pterygium Excision, Recurence, iGen, limbal Auto-graft, Subconjunctival Implantation

## Abstract

This study was conducted to evaluate the effect of collagen matrix implant on pterygium recurrence compared with a control group following pterygium removal surgery. Following informed consent, twenty eyes of 20 patients (females = 13, males = 7, aged 23–67 years) were randomly allocated into two equal groups. Pterygia of all patients were excised using the bare sclera technique; however, for patients in the intervention group a 1×2 mm collagen matrix graft (iGen™) was implanted subconjunctivally. Patients were followed up for recurrence and complications within six months. Results revealed that only two eyes in the control group and one eye in the intervention group developed recurrence (p=0.50); no dellen formation was observed. In conclusion, the use of biodegradable collagen matrix implants (iGen™) following pterygium excision seems to be associated with a lower risk of recurrence; however, the statistical difference between groups was not significant. Further studies with adequate sample size are recommended.

## INTRODUCTION

Pterygium is a common disorder in many parts of the world, with reported prevalence rates ranging from 0.3 to 29%. Epidemiological studies have proposed an association with chronic exposure to sunlight and an increased geographical prevalence within a peri-equatorial ‘pterygium belt’ region (1). While there is currently no universal consensus for grading of pterygium, one of the current methods has graded pterygia as follows: stage 0: pinguecula posterior to the limbus; stage 1: the pterygium is restricted to the limbus; stage 2: the pterygium only marginally invades the cornea; stage 3: the pterygium is between the limbus and pupillary margin; and stage 4: the pterygium is central to the pupillary margin (2).

 While pterygium excision is often combined with various adjunctive measures to prevent its recurrence (1), this is still the most common post-surgical complication and represents a significant surgical problem. Numerous techniques have been attempted to reduce localized fibrovascular activity and the overall rate of recurrence; these include, β-irradiation, conjunctival and limbal auto-grafting, antimitotic drugs and amniotic membrane transplantation (3).

 In recent years, a novel bioengineered product has been suggested and has shown promising outcomes in animal models (4). Biodegradable collagen matrix implants (iGen™) are porous scaffolds that can encourage a regenerative and non-scarring wound healing process without the use of medications. Its manufacturer has described its use not only for trabeculectomy, but also for other ophthalmic surgeries such as pterygium removal, oculoplastic surgery and veterinary surgery (5). The principle of the iGen™ collagen implant is to stimulate random growth of fibroblasts, which subsequently leads to normal wound healing. The iGen™ scaffold’s porous structure can work as a reservoir, a buffering system, and also as a controlled drainage solution. It randomizes the growth of myofibroblasts and creates a novel physiological environment between the anterior chamber and subconjunctival space of the eye. It maintains the physiological barrier by regeneration, which then maintains both the function of the bleb and normalizes the dynamic aqueous balance to create a safe conjunctival system. It also prevents scar formation inside the wound by guiding fibroblasts to grow randomly throughout the matrix pores (4,5). In this study, we aimed to evaluate post operation complications of iGen™ in pterygium surgery.

## METHODS

In this prospective study we studied 20 eyes of 20 patients with pterygium who had been referred to Al-Zahra Ophthalmologic Hospital, Zahedan, Iran between January 2011 and March 2012. Patients were randomly assigned equally to either the intervention or control groups. Patients were included if they had pterygium in stage 3 measuring 3 mm and 2 mm in horizontal and vertical diameters, respectively, which were associated with recurrent red eye, foreign body sensation, active vascularized advancing head, decreased visual acuity due to involvement of visual axis, and cosmetic problems. Exclusion criteria included patients who had any history of ocular surgery including pterygium removal, presence of symblepharon, evidence of systemic diseases (e.g. diabetes, AIDS, hepatitis, etc.), past history of atopy and hypersensitivity, or traumatic injury to the anterior segment of the eye leading to surgical repair. This study was approved by ethical committee of Zahedan University of Medical Sciences, Zahedan, Iran and proper informed consents were obtained from patients.

 All patients underwent surgery using the bare sclera method. Following administration of topical and local anesthesia and placing the speculum in situ, the pterygium was removed in a sterile fashion. Prior to suturing the conjunctiva using 10-0 nylon or 7-0 Vicryl™, a 1×2 or 2×2 square millimeter porous collagen matrix (iGen™, Life Spring Biotech Co, Taiwan) was implanted beneath the conjunctiva of those in the intervention group. Twenty-four hours following surgery all patients in both groups received treatment with drops of ciprofloxacin and betamethasone four times a day for 1 week’s duration.

 Follow-up visits for evaluation of complications or recurrence were on the first day, first week, first month, third month and sixth month postoperatively. The presence of dellen was considered as a complication, and recurrence was defined as at least 1 mm regrowth of the conjunctival fibrovascular tissue toward the cornea in both vertical and horizontal diameters during the follow-up time and, in such cases, its size was calculated from advancing edge in mm2 (6). Absence of recurrence and/or complications was considered as indicating the efficacy or safety of the procedure. Subsequently, Fisher’s exact test was used in order to determine the statistical significance.

## RESULTS

Twenty eyes of 20 patients with unilateral or bilateral pterygium were enrolled (females = 13 females, males = 7, aged 23–67 years). In the intervention group there was one patient who developed mild symptoms of recurrent inflammation, tearing, and foreign body sensation at the first month, which worsened in the third month following collagen implant with development of specified recurrence criteria. In the control group only two patients mentioned symptoms more prominently in the third month following surgery with recurrence of pterygium noted on slit lamp examination. However, there were no complications such as dellen formation in the follow-up visits in both groups.

## DISCUSSION

We compared the effects of subconjunctival implantation of a collagen matrix (iGen™) following pterygium removal by the bare sclera method in an intervention group with a control group who were treated solely with bare sclera method and found a higher rate of recurrence among the latter. The statistical difference, however, was not significant (p>0.05) and should be attributed to the small sample size and short duration of follow up.

 Pterygium is one of the most common conjuctival disorders, especially evident in regions with high ultraviolet exposure and a dry climate (3,7) such as Zahedan in South East, Iran.

 Despite the introduction of several techniques for treatment of pterygium, recurrence is still an ophthalmologic dilemma. The definition of pterygium recurrence, however, varies among studies. Most ophthalmologists define pterygium recurrence as regrowth of fibrovascular pterygium-like tissue crossing the limbus on to the cornea, fibrovascular recurrence to the same degree of corneal encroachment as the original lesion, or regrowth exceeding 1 mm onto the cornea (1). There are many options for wound closure after pterygium removal such as simple closure, rotational or sliding flap, free flap and/or limbal transplantation (7-9).

In a non-randomized trial, Madrazo et al. deliberated the efficacy of a biodegradable collagen matrix implant (OculusGen®) following pterygium excision on 20 eyes, and followed up the patients for at least 3 months. They reported a pterygium recurrence rate of 5% without any complications or adverse effects (10). In general, biodegradable collagen matrix implants have been used safely following trabeculectomy and they seem to be used as alternating treatments with antimitotic drugs (5,11); however, there are few reports of their efficacy in preventing pterygium recurrence. One of the limitations in our study was the bare sclera technique used for pterygium removal as this technique is less commonly used.

 In conclusion, the implantation of collagen matrix is a quick and easy technique, may be associated with lower rate of pterygium recurrence and subsequently may improve outcomes from the bare sclera method of surgery. Further studies with a larger sample size and longer duration of follow up are recommended to further explore this technique.
